# Diagnostic value of death certificates for case ascertainment of dementia: A population-based assessment of mortality registry data

**DOI:** 10.1177/13872877261423958

**Published:** 2026-02-25

**Authors:** Frank J. Wolters, Peter P.M. Harteloh, Frank J.A. van Rooij, Pieter Bakx, Jolande van Heemst, Brenda C.T. Kieboom, M. Arfan Ikram

**Affiliations:** 1 Department of Epidemiology, Erasmus MC, Rotterdam, the Netherlands; 2 Department of Radiology & Nuclear Medicine, Erasmus MC, Rotterdam, the Netherlands; 3 8220 Department of Health and Care, Statistics Netherlands, The Hague, the Netherlands; 4 Erasmus School of Health Policy & Management, 6984 Erasmus University Rotterdam, Rotterdam, Netherlands; 5 Department of General Practice, Erasmus MC, Rotterdam, the Netherlands

**Keywords:** Alzheimer's disease, cause-of-death, dementia, diagnostic value, epidemiology, mortality coding, registry

## Abstract

**Background:**

Use of mortality registry data could facilitate dementia case ascertainment in research, but its diagnostic value is uncertain.

**Objective:**

To determine the diagnostic value of death certificates for ascertainment of dementia.

**Methods:**

We included 6280 deceased participants of the population-based Rotterdam Study (mean age at death: 83.6 years, 57% women), who had been actively followed during life for the occurrence of dementia through repeated in-person and medical record screening between 1989–2018. We compared lifetime dementia diagnosis to dementia being certified as cause-of-death in the Dutch national mortality registry. We determined concordance of death certificates according to coding method, place of death, and participant characteristics (age at death, dementia duration, and comorbidity). To validate observations, we surveyed 446 certifying physicians.

**Results:**

Overall, 1828 persons (29%) had a diagnosis of dementia during life. Specificity of death certificates for dementia diagnosis consistently exceeded 96%. Sensitivity was below 10% using ICD-9 but increased to about 70% in more recent years using ICD-10. Diagnostic accuracy improved with introduction of ICD-10 (odds ratio [95%CI]: 1.94 [1.08–3.48]), and varied substantially with age at death (OR per decade increase: 0.80 [0.66–0.96]), dementia disease duration (OR [95%CI] for ≥6 years versus 0–2 years: 3.95 [2.56–6.09]), place of death, and comorbidity (OR [95%CI] per additional illness: 0.82 [0.72–0.93]), particularly due to stroke or heart failure. Dementia severity and duration were also reported by physicians as important factors for certifying dementia as a cause of death.

**Conclusions:**

Death certificates have high specificity but limited sensitivity for diagnosis of dementia, in particular in older patients with cardiovascular comorbidity.

## Introduction

Accurate ascertainment of dementia in longitudinal cohort studies is important to obtain unbiased answers to research questions and generally involves a strenuous and costly combination of repeated in-person examinations and medical records screening. These thorough methods for case ascertainment, however, are often not feasible in contemporary biobanks, with population sizes exceeding 100,000 participants. Consequently, these biobank cohorts increasingly rely on registry data for dementia diagnosis, including clinical diagnostic coding, claims data, and mortality registries. Moreover, an increasing number of registry-based studies uses exclusively routinely collected healthcare data for dementia diagnosis. The validity of these methods for case ascertainment depends in part on the diagnostic value of the registry-based data. Death certificates are an important source of routinely collected healthcare data, used for detecting occurrence of dementia in study populations. As such, it is important to know how well the diagnosis of dementia mentioned on the death certificate corresponds to a clinical diagnosis made during life.

A 2014 systematic review of 7 population-based studies concluded that the use of death certificates for studying dementia grossly underestimates dementia occurrence in the population.^1^ Yet, the source reports relied for a large part on data from the 1990s. The diagnostic value of cause-of-death coding has changed over time, with revised editions of the International Statistical Classification of Diseases (ICD).^2^ Indeed, the UK Cognitive Function and Ageing Study (CFAS) more recently reported an increase in the sensitivity of death certificates for dementia, from 21% in the period from 1989–2007 to 45% between 2008–2016. This came at the expense of a small drop in specificity from 99.5 to 97.9%.^3^ While part of these trends may be attributable to the introduction of ICD-10 around 1994, it is unknown to which extent other temporal trends in certification played a role, especially in view of the automatic system for coding multiple causes of death that was introduced and endorsed by the World Health Organization in 2017 (i.e., implemented by IRIS software).^4^ Moreover, reported accuracy of death certificates varies substantially in contemporary literature, with sensitivity ranging from 45% in CFAS to 63% over a similar time period in the Swedish Dementia Registry (2007–2012).^3,5^ These differences could be attributable to preferential registration of more severe dementia cases in both the Swedish registry and death certificates, but may also relate to differences in other patient and disease characteristics, including comorbidity.

Reporting of dementia on death certificates in two studies from the UK was more frequent with severe compared to earlier stage dementia,^3,6^ while evidence is inconclusive on the impact of place of death (e.g., at home or in hospital).^3,6^ Specificity was not investigated. Similarly in Spain, severity of dementia and age at death were related to sensitivity of death certificates.^7^ Across countries dementia type has been observed to influence reporting.^5–7^ Moreover, sensitivity of death certificates for dementia diagnosis has been reported lower in patients with respiratory and circulatory diseases,^1^ as well as Parkinson's disease.^8,9^ Such variation could induce differential misclassification in studies that use death certificates as the only means of dementia case ascertainment, but the magnitude and therefore relevance of such mechanisms with contemporary cause-of-death coding are undetermined.

We therefore compared reporting of dementia on death certificates in a Dutch national cause-of-death registry, with prospective, lifetime diagnosis of dementia in an ongoing population-based cohort study with active case-ascertainment. We determined temporal trends in diagnostic accuracy of death certificates and assessed the impact of several patient and disease characteristics on cause-of-death reporting.

## Methods

### Rotterdam study population

This study is embedded within the Rotterdam Study, a large ongoing population-based cohort study in the Netherlands among individuals aged ≥45 years residing in the Ommoord area, a suburb of Rotterdam. The Rotterdam Study methods have been described in detail previously.^10^ For the present study, we included all individuals who had completed screening for dementia at cohort inception since 1990 and passed away before 1 January 2018. The Rotterdam Study has been approved by the medical ethics committee according to the Population Screening Act Rotterdam Study, executed by the Ministry of Health, Welfare and Sports of the Netherlands. Written informed consent is obtained from all participants at each center visit. Due to data sharing restrictions in part of the informed consents (i.e., an earlier version of the form), we could complete linkage of the study database with the national death registry for 6947 of 7816 eligible participants (89%), of whom 6280 had completed cognitive screening at baseline and were dementia-free.

### Dementia screening and surveillance

Participants of the Rotterdam Study were interviewed at home and examined at a dedicated research center every 4 years since cohort inception.^10^ Participants were screened for dementia at baseline and subsequent examination rounds with the Mini-Mental State Examination and the Geriatric Mental Schedule organic level. Those with a Mini-Mental State Examination score <26 or Geriatric Mental Schedule score >0 underwent further investigation and informant interview, including the Cambridge Examination for Mental Disorders of the Elderly. At each center visit, all participants also underwent routine cognitive assessment, including a verbal fluency test (animal categories), 15-word learning test, letter-digit substitution task, Stroop test, and Purdue pegboard task. In addition, the entire cohort was continuously under surveillance for dementia through electronic linkage of the study center with medical records from general practitioners and the regional institute for outpatient mental health care. Available clinical neuroimaging data were reviewed when required for diagnosis of dementia subtype. A consensus panel headed by a consultant neurologist established the final diagnosis according to standard criteria for dementia (DSM-III-R). Follow-up was near-complete, with only 244 (5.5%) participants being censored for loss to follow-up prior to death.

### Mortality ascertainment and cause of death

In routine follow-up of the Rotterdam Study cohort, information on vital status was obtained through a bimonthly check of municipal records. Follow-up for mortality until 1 January 2018 was virtually complete. Place of death was obtained from municipal records. Other than date and place of death, and what was reported in the patients’ medical notes, no formal death certificate data were used in routine data collection for the Rotterdam Study. For participants that had passed away before 1 January 2018, we requested mortality data from Statistics Netherlands.

In the Netherlands, for every deceased person a death certificate is completed by the attending physician and sent to Statistics Netherlands for coding and the production of cause-of-death statistics. The attending physician is asked to report the causal chain of morbid events leading to death (i.e., part 1 of the death certificate) and (if applicable) diseases or disorders acting as contributory cause(s) of death while not being part of the causal chain (i.e., part 2 of the death certificate). Treating physicians may have been aware of their patient's participation in the Rotterdam Study cohort, but were never prompted by the study team to adapt their clinical routines, and there have been no prior Rotterdam Study reports that could have raised awareness among physicians about cause-of-death certification. All death certificates are coded and stored anonymously by Statistics Netherlands. Coded death certificates are available on request for research purpose by academic hospitals and research institutes.

From 1989 to 1995, coding was based on the International Classification of Diseases, 9th revision (ICD-9), after which this was replaced by the ICD-10. Dementia was defined based on specific codes from the ICD-9 (290.0–4, 290.8–9, 291.1–2, 294.0–1, 331.0–9, 438.0) and ICD-10 (F00–04 and G30-31). From 2013 onwards, the ICD-10 coding was partly automated using IRIS, an internationally applied electronic system for coding of causes of death.^4,12^ The switch to IRIS implied that Statistics Netherlands could no longer share secondary, contributing causes of death with external parties as of 2013. Hence, from 2013 onwards, we had data available only for the primary cause of death of the Rotterdam Study participants, which we correct for statistically (please see the “statistical analysis” section for details).^14^

### Survey among certifying physicians

To obtain insight in the reasons for certifying dementia not otherwise specified (ICD-10: F03) as a cause of death, we sent out a survey to a random sample of 700 physicians in the Netherlands, within four months after they had mentioned “dementia” on a death certificate in the second half of 2017.^11^ The corresponding population of deceased persons comprised 31% men and 69% women, with a mean age at death of 87 years. Questions used for this paper were part of a broader survey,^11^ of which three questions where intended to assess reasons for certifying dementia on the death certificate (i.e., duration, severity, and age at death). Physicians were asked to respond on a 7-point Likert scale. The precise questions are reported in Supplemental Table 1. Of 700 invitees, 446 physicians filled in the survey (65%). Age and gender distributions of their deceased patients were similar to those of the sampling frame.

### Analysis

Analysis included all deceased participants who had undergone screening for dementia at cohort inception. First, we compared dementia diagnoses from death certificates to all-cause dementia diagnosis in the Rotterdam Study, and stratified by year of death. Assuming the cohort lifetime diagnosis as gold standard, diagnostic accuracy was defined as the number of true positives and true negatives, divided by the overall sample size. We then assessed time trends and the impact of changes in coding methods on diagnostic accuracy, accounting for age at death and dementia disease duration by means of segmented regression analysis, breaking down time to 6-month segments and using 1 January 1996 and 1 January 2013 as change points.^13^ These time-series models included participant level data, and therefore only included primary causes of death during the IRIS period from 2013 onwards. Due to the lack of available data on secondary, contributing causes of death after 2013 (i.e., the IRIS timeframe), we calculated a corrected sensitivity to deal with this missing information and ensure comparability over time, by weighing the observed sensitivity by $\frac{\mathrm{COD}2\;\mathrm{dementia}}{\mathrm{COD}1\;\mathrm{dementia}}$ , in which COD1_dementia_ reflects the number of death certificates with dementia as primary cause of death and COD2_dementia_ reflects certificates with dementia as contributing cause of death, as reported to Statistics Netherlands across the entire country between 2013–2018.^14^

We then investigated determinants of diagnostic accuracy, focusing on age at death, dementia characteristics, comorbidity at time of death, and place of death (i.e., hospital, home, nursing home). Duration from dementia diagnosis till death was taken as a proxy for dementia severity at time of death. Comorbidity included disease entities with substantial effect on wellbeing and life-expectancy, for which detailed, continuous follow-up data were available in the Rotterdam Study: coronary heart disease, heart failure, stroke, chronic obstructive pulmonary disease, and cancer (excluding non-melanoma skin cancer).^15–19^ To assure applicability of our findings to present-day coding, we restricted these analyses to the ICD-10 era (pre-IRIS).

Analyses were performed using IBM SPSS Statistics, version 28.0 (IBM Corp, Armonk, New York). The α (type 1 error) was set at 0.05.

## Results

A total of 6280 deceased participants were included, of whom 3604 (57%) were women. Mean age at death was 83.6 years (standard deviation: 8.7 years). Among 5906 individuals with known place of death, most people passed away in hospital (30.4%), followed by nursing homes (27.6%), at home (23.8%), in a residential care home (15.7%), or elsewhere (2.5%).

### Agreement between death certificates and diagnosis during lifetime

Overall, 962 participants (15.3%) had dementia noted on their death certificate, either as primary or contributing cause of death. In comparison, on the basis of dementia surveillance in the Rotterdam Study, dementia was diagnosed in 1828 (29.1%) of participants during their lifetime. The vast majority of discrepancies between lifetime diagnosis and death certificate concerned 962 cases in whom lifetime dementia diagnosis was not recorded as a cause of death. However, for 96 others, dementia was listed among the causes of death for individuals who were not considered to have dementia by the expert review panel based on earlier in-person screening and medical records check. In half of cases (45/96; 47%), this was due to apparent errors in mortality coding, as there was no evidence of a clinical neurocognitive disorder in the cohort data. In 18 others (19%), symptoms recorded in the cohort data were suggestive of dementia, but guidelines precluded a formal diagnosis due for instance to psychiatric comorbidity, recent delirium, or insufficient cognitive assessment. In two cases there was clear evidence of clinical dementia in medical records that was missed during record screening. A further 8 participants were diagnosed with mild cognitive impairment during life, without repeated assessment for disease progression in their remaining lifetime (range: 0.1–5.4 years). The remaining 23 (24%) participants were lost to follow-up for in-person screening and medical records check; these are discussed in more detail in the before last paragraph of the results section.

### Diagnostic value according to coding method and place of death

**Figure 1.**
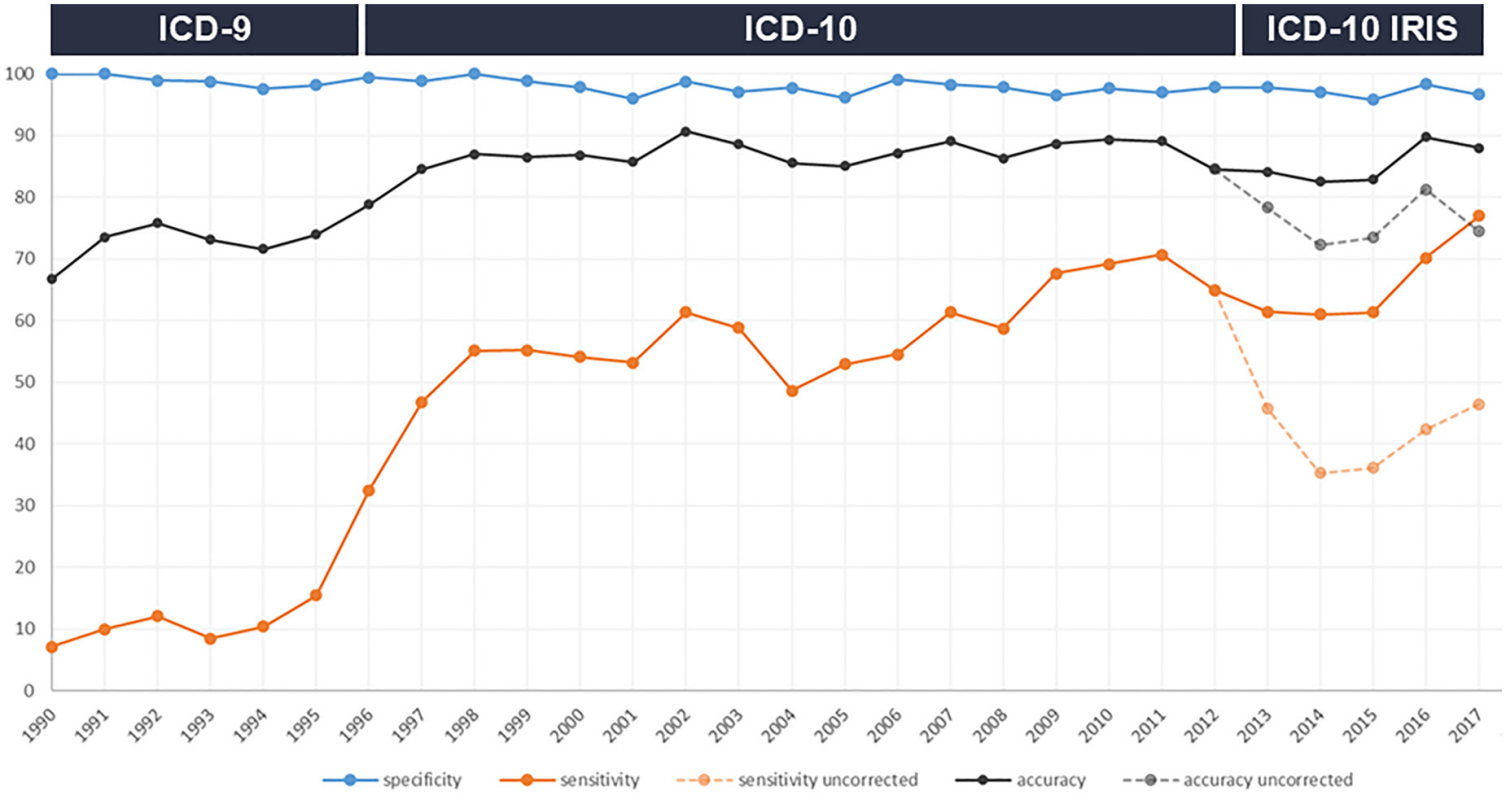
Diagnostic value of death certificates for the diagnosis of dementia over time. Sensitivity, specificity, and accuracy by year, in relation to changes in ICD coding for national death statistics. Diagnostic value during the IRIS period has been corrected using group level data, as individual participant level data on contributing causes of death was unavailable between 2013–2017 (details are provided in the methods section).

Diagnostic accuracy of cause-of-death coding for dementia improved over time, from around 70% in the early 1990s to close to 90% in more recent years (Figure 1). Specificity was stable >96%, whereas sensitivity varied substantially depending on coding method. Improvements in diagnostic accuracy were mostly related to introduction of the ICD-10 (OR: 1.94, 95% CI: 1.08–3.48; Table 1), and remained stable thereafter when using complete IRIS data (Figure 1). Following introduction of the ICD-10 (prior to IRIS), sensitivity and specificity were 56.8% and 97.9%, respectively, with an accuracy of 87%. Agreement between lifetime diagnosis and cause-of-death coding was much lower when using the primary cause of death only (Figure 1).

**Table 1.** Time trends in the diagnostic accuracy of death certificates, in relation to mortality coding and place of death.

	Model 1			Model 2	
Coding method	OR (95% CI)	*p*		OR (95% CI)	*p*
Trend during ICD-9	1.01 (0.96–1.06)	0.752		0.89 (0.77–1.02)	0.103
Introduction ICD-10	2.44 (1.81–3.29)	<0.001		1.94 (1.08–3.48)	0.026
Trend during ICD-10	0.99 (0.95–1.04)	0.750		1.14 (0.99–1.31)	0.069
Introduction IRIS*	0.42 (0.29–0.62)	<0.001		0.30 (0.18–0.51)	<0.001
Trend since IRIS	1.02 (0.96–1.09)	0.485		1.02 (0.94–1.11)	0.616

Effect estimates are derived from a segmented time-series regression model. The estimates for coding methods reflect the relative odds of correct diagnosis associated with the introduction of a new method, or per time unit increase (i.e., 6 months). Data during the IRIS period reflect the “uncorrected” dashed line presented in Figure 1, as during this period individual participant level data for contributing causes of death were not available for the time-series models. As can be appreciated from Figure 1, introduction of IRIS did not influence coding accuracy when incorporating primary as well as contributing causes of death.

OR: odds ratio; CI: confidence interval. Trend during ICD-9 is the baseline trend. Model 1 is the crude analysis. In model 2, analyses were further adjusted for place of death, age at death, sex, and dementia disease duration.

* Data during the IRIS period reflect the “uncorrected” dashed line presented in Figure 1, as during this period individual participant level data for contributing causes of death were not available for the time-series models. As can be appreciated from Figure 1, introduction of IRIS did not influence coding accuracy when incorporating primary as well as contributing causes of death.

**Figure 2.**
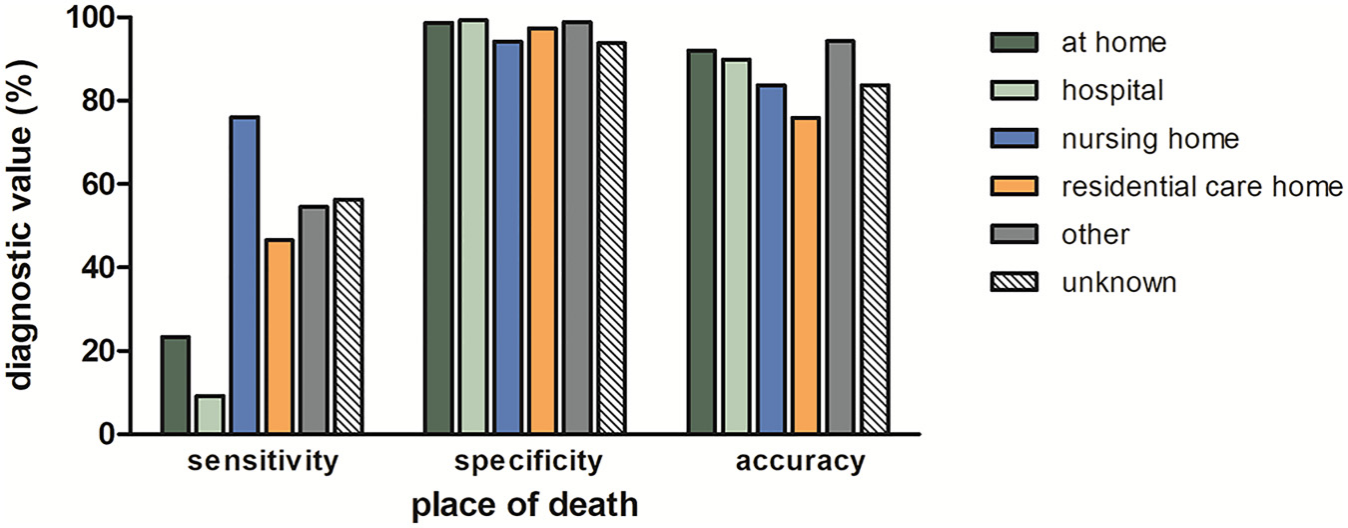
Diagnostic value of death certificates in relation to place of death. Bars represent the sensitivity, specificity, and accuracy of certificates for cases occurring within the ICD-10 manually coded period. Accuracy can be derived from the sensitivity, specificity, and prevalence of disease (i.e., accuracy = sensitivity * prevalence + specificity * (1-prevalence). Sensitivity was lowest for cases who died at home or in hospital, but these locations yielded a higher overall accuracy owing to higher specificity and lower prevalence of dementia, as compared to participants residing in nursing homes or residential care homes at time of death. Sample sizes were as follows: at home (n = 1405; 22.4%), in hospital (n = 1794; 28.6%), in a nursing home (n = 1631; 26.0%), in a residential care home (n = 926; 14.7%), elsewhere (n = 150; 2.4%), and unknown place of death (n = 374; 6.0%).

Individuals with dementia died more often in nursing homes (55.6%) or residential care homes (23.9%) than those without dementia (16.1% and 12.3%, respectively). Only 7.9% of people with dementia died at home (135/1719, versus 1270/4187 [30.3%] dementia-free) and 11.8% died in hospital (202/1719, versus 1592/4187 [38.0%] dementia-free). Sensitivity of death certificates for dementia was highest for people who passed away in a nursing home, followed by those in residential care homes (Figure 2). However, overall accuracy in these care settings was lower than for people who died at home or in hospital, owing to a slightly lower specificity and much higher dementia prevalence in nursing homes and residential care homes, as compared to participants residing at home or being admitted to hospital at time of death (Figure 2).

### Determinants of diagnostic accuracy

Diagnostic accuracy of death certificates increased with longer disease duration, from 40.9% with diagnosis within 2 years before passing to 75.4% with a diagnosis of >6 years prior to death (Supplemental Table 2). Older age at death was also associated with a lower odds of accurately reporting dementia on the death certificate (Table 2), due predominantly due a slightly lower specificity after age 80 (Supplemental Table 2). Accuracy was lower in women than in men (OR 0.58 [0.48–0.70]), but this attenuated and was no longer statistically significant after adjustment notably for age at death and disease duration (OR 0.85 [0.65–1.11], Table 2). A higher number of concurrent illnesses at time of death was associated with a lower diagnostic accuracy of certificates for dementia (OR [95% CI] per additional comorbid illness: 0.82 [0.72–0.93], p = 0.002). This was driven by a lower likelihood of dementia being mentioned among the causes of death in people with congestive heart failure or prior stroke (Table 2). Once again, the difference was mostly attributable to lower specificity in the presence of these comorbidities (Supplemental Table 2).

**Table 2.** Impact of participant and disease characteristics on diagnostic accuracy.

	Univariable analysis		Multivariable analysis	
Determinant	OR (95% CI)	p	OR (95% CI)	p
Age at death (per 10-year increase)	0.49 (0.44–0.55)	<0.001	0.80 (0.66–0.96)	0.016
Female sex	0.58 (0.48–0.70)	<0.001	0.85 (0.65–1.11)	0.235
Dementia disease duration				
0–1.9 years	Reference		Reference	
2.0–3.9 years	1.60 (1.16–2.20)	0.004	1.66 (1.17–2.36)	0.005
4.0–5.9 years	3.11 (2.15–4.51)	<0.001	3.36 (2.23–5.06)	<0.001
≥6 years	4.42 (2.97–6.58)	<0.001	3.95 (2.56–6.09)	<0.001
Comorbidity				
Stroke	0.63 (0.52–0.77)	<0.001	0.71 (0.54–0.93)	0.012
Coronary heart disease	1.14 (0.94–1.39)	0.18	0.98 (0.75–1.30)	0.905
Congestive heart failure	0.80 (0.66–0.98)	0.027	0.61 (0.46–0.81)	<0.001
Chronic obstructive pulmonary disease	1.66 (1.26–2.18)	<0.001	0.86 (0.59–1.26)	0.438
Cancer	3.58 (2.78–4.60)	<0.001	1.33 (0.94–1.88)	0.103
Place of death				
At home	Reference		Reference	
Hospital	0.77 (0.58–1.03)	0.074	1.05 (0.69–1.61)	0.814
Nursing home	0.44 (0.34–0.58)	<0.001	4.29 (2.82–6.52)	<0.001
Residential care home	0.27 (0.20–0.36)	<0.001	1.60 (1.01–2.53)	0.045
Other	1.44 (0.61–3.38)	0.404	2.01 (0.67–5.99)	0.210
Unknown	0.44 (0.30–0.66)	<0.001	1.27 (0.70–2.30)	0.432

Odds ratios reflect the association of age and place of death, sex, disease duration, and comorbidity on the likelihood of an accurate dementia diagnosis on the death certificate. The models including dementia disease duration further included a dummy duration variable for non-demented participants. The difference between the univariable and multivariable models in estimates for place of death were due predominantly to adjustment for dementia disease duration. For the disease duration models, we excluded 183 participants with prevalent dementia whose precise time since diagnosis was unknown.

OR: odds ratio; CI: confidence interval. Analyses are restricted to the ICD-10 era (pre-IRIS).

### Potential use of registry data for compensating loss to follow-up

Of all 4452 participants who were not diagnosed with dementia during their lifetime, 244 (5.5%) were lost to follow-up at some point prior to death. This included individuals who had had repeated cognitive assessment, but with insufficient information to be certain about their dementia status from that moment onwards. Among these 244 individuals, censoring occurred a median 2.8 years prior to death (interquartile range: 1.2–5.3 years). As mentioned above, 9.4% of these individuals (23/244) had dementia reported among their causes of death. The interval between loss to follow-up and death was longer for those with dementia as a cause of death, than for those without dementia on their death certificate (median 6.5 [4.4–10.5] versus 2.5 [1.1–4.5] years, p-value<0.001).

### Survey among certifying physicians

Of 446 medical doctors who completed the survey, most were specialist in care for older patients (335/446; 75%) or general practitioner (83/446; 19%). The majority (85%) had been practicing for over 5 years at time of survey, and most had extensive experience in filling in death certificates (median of 20 per year). About half of certifying doctors had had contact with the deceased prior to his or her death (254/442; 58%), but only a small minority had established the diagnosis of dementia themselves (23/423; 5%), mostly relying on diagnosis by a geriatrician (n = 88), GP (n = 33), elderly care physician (n = 41), psychiatrist (n = 15) or neurologist (n = 15).

**Figure 3.**
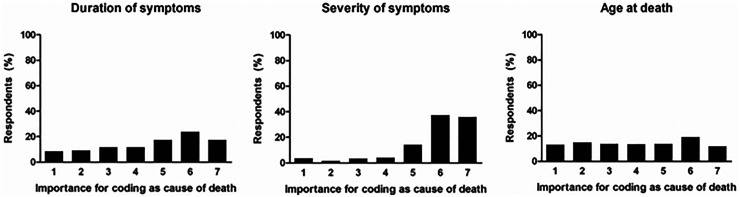
Reasons for certifying dementia as a cause of death. Results from a survey among 446 medical doctors who certified dementia as cause of death on a death certificate. Respondents indicated the importance of each of three parameters, from unimportant (1) to highly important (7).

Three quarters of physicians stated that dementia was an important or very important factor relating to death (332/439; 76%), in line with 58% of physicians certifying dementia as the primary underlying cause of death (221/384 with valid data). Severity of symptoms, and to a lesser extent duration of symptoms, were marked as an important reason for reporting dementia on the death certificate (Figure 3). Among this sample of physicians who certified dementia among the causes of death, age did not play a role in their decision for doing so (Figure 3).

## Discussion

In this population-based study with detailed, prospective case ascertainment of dementia, we observed high specificity but limited sensitivity of nationwide mortality registry for the diagnosis of dementia. When incorporating both primary and contributing causes of death, diagnostic accuracy of death certificates has improved greatly with the introduction of ICD-10, but still varies substantially with age at death, place of death, dementia characteristics, and cardiovascular comorbidity at time of death.

The sensitivity of death certificates for reflecting lifetime clinical diagnosis of dementia in the current study is in line with results from the Swedish Dementia Registry, and somewhat higher than reported in the Cognitive Function and Ageing Study (CFAS) in the UK.^3,5^ We observed no temporal trends in the reporting of dementia on death certificates, above and beyond the changes in coding system. The observed level of sensitivity implies that mortality registry data by itself are insufficient to reliably quantify occurrence of dementia, even when using multilevel cause-of-death registration. Limited sensitivity reflects, at least in part, the sensible requirement in WHO guidelines that contributing causes of death need to be part of the causal chain. The task of the certifier is to selected diseases or disorders from the clinical history, that are present at time of death, according to their causal role in the passing of the patient. Therefore, the limited sensitivity of death certificates for capturing lifetime prevalence of dementia does not necessarily indicate an error in certification, but foremost points at the limited perceived role of dementia as cause of death. Whilst the more or less comparable findings across European countries support a certain generalizability of our results, observations may well differ in countries and cultures with markedly different healthcare systems or notions on health, disease and death.

Despite the increasing tendency to attribute death to a cluster of diseases or disorders, rather than a single underlying cause,^14^ additional data sources are needed beyond mortality registries, to reliable ascertain the vast majority of dementia cases through registry data. A combination of multiple datasets may increase diagnostic yield to detect dementia, as was previously shown for cancer diagnosis.^20^ Additional incorporation of pharmacy dispensing records, hospital records, aged care assessments, and claims data may capture an estimated three quarters of all dementia cases.^21,22^ Congruent information from multiple, different data sources could also increase diagnostic certainty, but death certificates agree poorly with claims data,^23^ and one third to half of dementia cases in aforementioned study was identified through a single source only.^21,22^ The share of these “single source cases” that stems from death certificates is uncertain, with numbers in aforementioned reports ranging between 4% and 43%.^21,22^ Further study is warranted to compare a diagnosis from multiple sources of routinely collected healthcare data, directly to case ascertainment that involves active screening through in-person examinations. Although specificity is consistently above 97%,^3^ it is useful to include estimates of specificity in such studies, as seemingly small variations in specificity can have substantial impact on diagnostic accuracy when prevalence of the disease is low in the population of interest, such as for early-onset dementia.

As demonstrated in the current study, the diagnostic value of a mortality registry for dementia depends strongly on patient and disease characteristics. Both our quantitative and qualitative analyses highlight dementia duration, or severity, as important determinants of whether dementia was reported on the death certificates. In line with an Australian study,^21^ we also found less frequent mentioning of dementia on death certificates for women than for men, but this was explained by later age at diagnosis and death for women. Age at death was an important factor in the quantitative analyses, irrespective of the measured comorbidities at time of death. This could indicate that doctors may consider some degree of cognitive decline “normal” to the process of aging. In line with this reasoning, the physicians in our survey who, by survey design, all reported dementia on the certificate of their older, deceased patient population, did not consider age to matter for reporting. Whether or not dementia was a contributing *cause* to a patient's death, of course, is inevitably subject to interpretation of the treating physician. The question whether the patient would have died in the absence of dementia is often hard to estimate as both medical (e.g., susceptibility to infections) and behavioral aspects (e.g., treatment compliance) play part. Hospital diagnoses are often skewed towards the acute cause of death, and the extent of prior cognitive of mental illness may not be immediately clear among treating physicians, or judged non-contributing to the passing of the patient.^24,25^ Similarly, general practitioners who certify the cause of death for a patient during out-of-office hours may not be fully aware of prior conditions. Indeed, our survey shows that only half of certifying physicians had known the patient during life, and good access to electronic patient records across healthcare settings therefore is crucial. Moreover, clear instructions to physicians on certifying dementia as a cause of death, both during their training and on the job, are essential to reduce heterogeneity due to interrater variability in these important nationwide statistics.^26^

If used as the only data source, poor accuracy of death certificates for ascertainment of lifetime disease causes underestimation of *absolute* risks, but, as long as misclassification of the outcome occurs at random, leaves *relative* risk estimates largely unaffected. In other words, low sensitivity or specificity for diagnosing dementia per se does not hamper study of disease determinants, as long as the diagnostic tool performs similar in the exposed and the unexposed individuals. However, we demonstrated that the presence of comorbidity influences whether or not certifying physicians report “dementia” as a cause of death. Especially in the context of stroke and heart failure, dementia may be overlooked or judged irrelevant to the death of a patient. The bias that arises from diagnostic misclassification in registry data could therefore distort in particular associations of dementia with heart failure or stroke (or risk factors thereof),^27^ but less so its association with for example cancer or chronic pulmonary disease. Studies of patients with late-onset dementia are likely to be affected more than those on early-onset dementia, due to higher prevalence of comorbidity.^28^ While the impact of such “competing” causes of death on study validity in present day and age warrants further study, we caution against the sole use of mortality statistics when investigating determinants that render adjudication of dementia as a cause of death less likely.

Strengths of this analysis include the meticulous follow-up for dementia in a population-based cohort over a prolonged period of time, and the ability to reliably assess a variety of parameters that may affect coding accuracy, including important comorbid illnesses. Certain limitations should be taken into account too. First, due to privacy regulations, we had limited participant-level data available from 2013 onwards on the IRIS coding, which we were able to adjust for on a population level only. More detailed participant-level data on contributing causes of data are warranted in future studies to provide a more fine-grained insight in the impact of IRIS on diagnostic accuracy in recent years. Second, the more recent introduction of the ICD-11 classification in 2022 was beyond the temporal scope of our data linkage. For the coming years, most longitudinal research will still make use of data collected at the time of ICD-10, but the impact of ICD-11 on coding accuracy nevertheless warrants closer inspection in future investigations. IRIS has been updated to accommodate for ICD-11 coding,^4^ but implementation of the system is pending and will require evaluation in future research. Third, despite rigorous assessment we might have missed a diagnosis of dementia in some participants of the Rotterdam Study, which would have led to overestimation of specificity. However, the small number of participants with a diagnosis on death certificates but not during in vivo Rotterdam Study participation supports the overall validity of our conclusions. Fourth, we had no consistent information on dementia severity at time of clinical diagnosis. Although the profound associations with time since diagnosis suggest that symptom severity is an important determinant of reporting dementia on a death certificate, we cannot directly infer this from our cohort analyses. Fifth, we only surveyed physicians who reported dementia on a recent death certificate, leading to underestimation in the survey of the importance of parameters with a strong *negative* influence on reporting. For example, mean age at death of patients whose physicians were invited for the survey was 87 years. Consequently, these physicians per inclusion had no age bias if it comes to reporting dementia, as reflected in Figure 3. As such, the apparent discrepancy between the survey and the Rotterdam Study observations with respect to age, likely reflects the selected recruitment of physicians into the survey.

In conclusion, death certificates have high specificity but limited sensitivity for diagnosis of dementia, in particular in elderly patients with recent diagnosis of dementia or with cardiovascular comorbidity. The low sensitivity reflects in part a limited perceived role of dementia as cause of death. These findings can serve to tailor instructions to certifying physicians. From a research perspective, our findings aid to decide in longitudinal studies when cause-of-death statistics may suffice to obtain valid relative risk estimates, and when to consult additional data sources such as in-person screenings or claims data.

## Supplemental Material

sj-docx-1-alz-10.1177_13872877261423958 - Supplemental material for Diagnostic value of death certificates for case ascertainment of dementia: A population-based assessment of mortality registry data Supplemental material, sj-docx-1-alz-10.1177_13872877261423958 for Diagnostic value of death certificates for case ascertainment of dementia: A population-based assessment of mortality registry data by Frank J. Wolters, Peter P.M. Harteloh, Frank J.A. van Rooij, Pieter Bakx, Jolande van Heemst, Brenda C.T. Kieboom and M. Arfan Ikram in Journal of Alzheimer's Disease
