# Supplementary material for: Diagnostic value of death certificates for case ascertainment of dementia: A population-based assessment of mortality registry data
Source: J Alzheimers Dis. 2026 Feb 25;110(3):1428–37. doi: 10.1177/13872877261423958 (PMC13022020; doi:10.1177/13872877261423958)
Supplement: sj-docx-1-alz-10.1177_13872877261423958 - Supplemental material for Diagnostic value of death certificates for case ascertainment of dementia: A population-based assessment of mortality registry data [file sj-docx-1-alz-10.1177_13872877261423958.docx]

# **Supplemental Material**

**Diagnostic value of death certificates for case ascertainment of dementia: A population-based assessment of mortality registry data**

**Supplemental Table 1.** Survey questions in the original Dutch version, and their English translation.

**Vraagstelling: Er kunnen verschillende redenen zijn om dementie te vermelden op het doodsoorzakenformulier. In welke mate speelden de volgende daarbij voor u een rol in dit overlijdensgeval?** Omcirkel het cijfer dat het beste past bij uw opvatting.


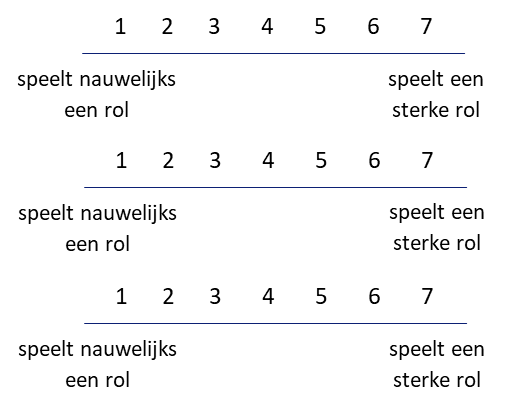


1. De ernst van de stoornis

2. De duur van de ziekte of aandoening

3. De leeftijd van de patiënt

**Question: There can be various reasons for reporting dementia on a death certificate. To which extent did the follow play a role in your decision to report dementia as a cause of death in this case?** Please encircle the number that best fits your views.


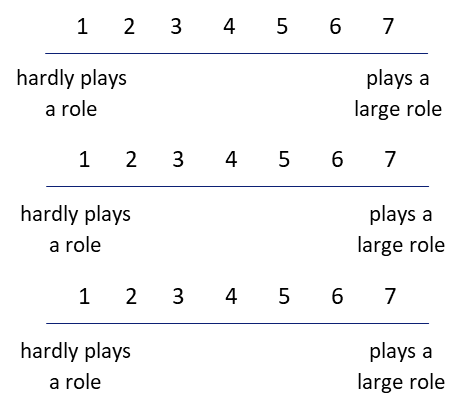


1. Severity of disease

2. Duration of disease

3. Age of the patient

**Supplemental Table 2.** Sensitivity and specificity of death certificates across patient groups and disease characteristics

|  |  | **Sensitivity** |  | **Specificity** | **Accuracy** |
| --- | --- | --- | --- | --- | --- |
| **Determinant** | **N_T+_/N_D+_** | **% (95% CI)** | **N_T-_/N_D-_** | **% (95% CI)** | **% (95% CI)** |
| Age at death |  |  |  |  |  |
| <70 years | 3/10 | 30.0 (16.0-58.4) | 358/358 | 100 (100-100) | 98.1 |
| 70-79 years | 83/156 | 53.2 (45.4-61.0) | 960/971 | 98.9 (98.2-99.6) | 92.6 |
| 80-89 years | 349/606 | 57.6 (53.7-61.5) | 1327/1361 | 97.5 (96.7-98.3) | 85.2 |
| ≥90 years | 259/449 | 57.7 (53.1-62.3) | 542/567 | 95.6 (93.9-97.3) | 78.9 |
| Sex |  |  |  |  |  |
| Female | 481/832 | 57.8 (54.4-61.1) | 1646/1699 | 96.9 (96.1-97.7) | 84.0 |
| Male | 213/389 | 54.8 (49.9-59.7) | 1541/1558 | 98.9 (98.4-99.4) | 90.1 |
| Dementia disease duration |  |  |  |  |  |
| 0-1.9 years | 115/281 | 40.9 (35.2-46.6) | n/a | n/a | n/a |
| 2.0-3.9 years | 175/333 | 52.6 (47.2-58.0) | n/a | n/a | n/a |
| 4.0-5.9 years | 151/221 | 68.3 (62.2-74.4) | n/a | n/a | n/a |
| ≥6 years | 153/203 | 75.4 (69.4-81.3) | n/a | n/a | n/a |
| Comorbidity |  |  |  |  |  |
| Stroke | 173/340 | 50.9 (45.6-56.2) | 686/704 | 97.4 (96.2-98.6) | 82.3 |
| Coronary heart disease | 138/286 | 48.3 (42.5-54.1) | 1059/1074 | 98.6 (97.9-99.3) | 88.0 |
| Congestive heart failure | 113/264 | 42.8 (36.8-48.8) | 824/842 | 97.9 (96.9-98.9) | 84.7 |
| Chronic obstructive pulmonary disease | 39/96 | 40.6 (30.1-50.4) | 606/613 | 98.9 (98.1-99.7) | 91.0 |
| Cancer | 70/137 | 51.1 (42.7-59.5) | 1247/1255 | 99.4 (99.0-99.8) | 94.6 |
| Place of death |  |  |  |  |  |
| At home | 21/90 | 23.3 (14.6-32.0) | 917/929 | 98.7 (98.0-99.4) | 92.0 |
| Hospital | 13/142 | 9.2 (4.5-13.9) | 1208/1216 | 99.3 (98.8-99.8) | 89.9 |
| Nursing home | 496/653 | 76.0 (72.7-79.3) | 451/479 | 94.2 (92.1-96.3) | 83.7 |
| Residential care home | 118/254 | 46.5 (40.4-52.6) | 337/346 | 97.4 (95.7-99.1) | 75.9 |
| Other | 6/11 | 54.5 (25.1-83.9) | 94/95 | 98.9 (96.8-100) | 94.3 |
| Unknown | 40/71 | 56.3 (44.8-67.8) | 180/192 | 93.8 (90.4-97.2) | 83.7 |

N: sample size; T+: test positive (dementia on death certificate); D+: disease positive (dementia during lifetime). Values are percentages. Analyses are restricted to the ICD-10 era (pre-IRIS).
